# Association of left ventricular global area strain derived from resting 3D speckle-tracking echocardiography and exercise capacity in individuals undergoing treadmill exercise test

**DOI:** 10.7150/ijms.75781

**Published:** 2022-09-11

**Authors:** Tsang-Wei Chang, Han-Chung Hsu, Wei-Chuan Tsai

**Affiliations:** 1 Division of General Internal Medicine, Department of Internal Medicine, National, Cheng Kung University Hospital, College of Medicine, National Cheng Kung University, Tainan, Taiwan.; 2 Division of Cardiovascular Medicine, Department of Internal Medicine, Kuo General, Hospital, Tainan, Taiwan.; 3 Division of Cardiovascular Medicine, Department of Internal Medicine, National, Cheng Kung University Hospital, College of Medicine, National Cheng Kung University, Tainan, Taiwan.

**Keywords:** global area strain, 3D speckle tracking echocardiography, exercise capacity

## Abstract

**Background:** Left ventricular (LV) global area strain (GAS) is a novel index derived from resting 3D speckle-tracking echocardiography (STE), and its clinical significance has rarely been studied. We examined the association of LV GAS and exercise capacity in a health check-up population.

**Methods:** We recruited 94 symptom-free participants (52.2 ± 11.7 years, 62.8% male) without substantial structural heart disease or coronary heart diseases who were undergoing a routine health examination. All participants underwent resting echocardiography and symptom-limited treadmill exercise test according to the Bruce protocol. Four strain parameters were obtained from the analysis, namely 3D GAS (GAS3d), global longitudinal strain, global circumferential strain, and global radial strain.

**Results:** After multivariate analysis for factors of exercise time, we observed a significant association in LV GAS3d (*P* < 0.001). We divided participants into preserved and impaired exercise capacity groups according to the cutoff value of 8 metabolic equivalent of tasks. LV GAS3d (OR 1.24, 95% CI 1.10-1.39, *P* < 0.001) was an independent predictor of impaired exercise capacity and the optimal cut-off value was -19.96% at a sensitivity of 77.8% and at a specificity of 92.1%. LV GAS3d could improve the discriminatory power of exercise capacity in individuals with early mitral filling velocity to average mitral annulus velocity ratio (E/e') ≥ 8.

**Conclusions:** LV GAS3d was significantly associated with exercise time and exhibited incremental predictive value on E/e' for exercise capacity in participants undergoing treadmill exercise test.

## Introduction

Three-dimensional speckle-tracking echocardiography (3D STE) is a recently developed imaging tool for left ventricular (LV) myocardial deformation analysis. It can precisely quantify LV strain by using complete 3D pyramidal data sets and has the potential to overcome the intrinsic limitations of two-dimensional STE (2D STE) 1, 2 such as through-plane motion of speckles. Compared with 2D STE, 3D STE has considerably faster image acquisition time and stronger reproducibility 3. Moreover, it offers a novel parameter known as global area strain (GAS), which is derived by LV endocardial surface area tracking and combines the effect of both longitudinal and circumferential shortening. GAS appears to exhibit the highest reproducibility of all 3D strain parameters 4. Three-dimensional STE has prompted advances in the diagnostic and prognostic evaluation of various cardiac diseases, including LV hypertrophy etiologies 5, 6, ischemic heart disease 7-9, heart failure 10, and valvular heart disease 11-13 as well as in cardiac surgery 14-16, cardio-oncology 17, 18, and cardiac resynchronization therapy 19, 20.

Exercise capacity can be determined using symptom-limited treadmill exercise test (TMT) and has been proven to be a prognostic factor in various disease conditions 21, 22. A recent study also demonstrated the relationship between STE and functional capacity 23; however, the association of echocardiographic parameters and exercise capacity has not been well elucidated. GAS, derived from 3D STE, is a new index of LV deformation. We hypothesized that LV GAS3d would play a crucial role in exercise capacity. Thus, the aim of this study was to determine the relationship between LV GAS3d and exercise capacity obtained by TMT in a health check-up population.

## Methods

### Study Population

From September 2011 to February 2013, a total of 96 individuals undergoing echocardiography and TMT in routine health examinations were recruited. In Taiwan, many people are willing to receive regular health check-up because of a belief that “prevention is better than cure” and the convenience of seeking medical treatment. All participants were free from symptoms and had no known structural heart disease, angiographically documented coronary artery disease, or atrial fibrillation. We carefully reviewed all participants' medical records, collecting the following information: clinical information on comorbidities, medical history, and current medication. Because of insufficient image quality for 3D STE analysis, two participants were excluded. Finally, 94 participants were enrolled in this study.

### Exercise Protocol

All participants underwent symptom-limited TMT according to the Bruce protocol 24. We recorded 12-lead ECG, blood pressure, and heart rate response in each 3-minute stage. If a patient exhibited moderate to severe angina, a decrease in systolic BP of more than 10 mm Hg, cyanosis or pallor, sustained ventricular tachycardia, ST elevation of 1.0 mm in non-infarct leads without diagnostic Q waves, or difficulty breathing, the treadmill test and recording were stopped. Similar to a previous report, we were able to use the metabolic equivalent of task (MET) system to explain participants' exercise capacity 25. The Goldman classification (Class I: >7 METs, Class II: 5-7 METs, Class III: 2-4 METs, and Class IV: <2 METs) 26, 27 is commonly employed in studies because it correlates with the New York Heart Association (NYHA) 28 functional classification criteria. However, in a previous study 25, it defined activities requiring an energy expenditure of 8 METs and above to be of high exercise intensity. In addition, a prospective study 29 of 6213 consecutive population referred for TMT confirmed the protective role of a higher exercise capacity even in the presence of other risk factors, especially for individuals whose exercise capacity was more than 8 METs. Thus, a MET of 8 is a reliable cutoff value and we defined preserved exercise capacity in this study as an energy expenditure of ≥8 METs.

### Echocardiography

Echocardiographic examinations and assessments were performed by independent echocardiologists who were blinded to participants' clinical information and TMT results. Standard resting 2D Doppler echocardiography (GE Vivid E9, GE Vingmed Ultrasound AS, Horten, Norway) was performed during the same week of the TMT. We evaluated the chamber size, LV systolic function, and LV diastolic function according to established guidelines 30, 31.

Three-dimensional full-volume acquisitions were made by a 4-V probe from an apical view, with volume rates of 20-40 volumes per second. Images were acquired from three cardiac cycles in sinus rhythm. The entire LV myocardium, including the epicardial surface, was captured within the pyramidal scan volume by appropriate adjustment of the sector width and depth. All acquired images were stored digitally for later offline analysis. All measurements were averaged over three cardiac cycles.

### STE Analysis

All 2D and 3D STE analyses were conducted using commercially available software (EchoPAC PC, version 113.1.1, GE Vingmed Ultrasound AS). Two-dimensional STE was performed using automatic function imaging for 2D global longitudinal strain (GLS2d), as described in our previous studies 32, 33. Three-dimensional STE analyses were undertaken to obtain 3D global longitudinal strain (GLS3d), global circumferential strain (GCS3d), global radial strain (GRS3d), and GAS (GAS3d) by using the 4D Auto Left Ventricular Quantification (LVQ) function in the software. The segments with suboptimal tracking were automatically rejected by the software. Only 0-2 segments were allowed unavailable in the analysis. Global strain values would not be calculated if equal or more than three segments were rejected.

### Reproducibility of parameters of 3D STE

We chose 20 random subjects for testing the variability. The intraclass correlation coefficient (ICC) of inter-observer and intra-observer reliability for GAS3d were 0.958 (95% CI, 0.889 to 0.983) and 0.989 (95% CI, 0.927 to 0.995), for GLS3d were 0.943 (95% CI, 0.862-0.977) and 0.967 (95% CI, 0.920-0.987), for GCS3d were 0.944 (95% CI, 0.864-0.977) and 0.962 (95% CI, 0.908-0.985), and for GRS3d were 0.931 (95% CI, 0.833-0.972) and 0.987 (95% CI, 0.968-0.995), respectively. The variability showed excellent agreement.

### Statistical Analysis

We used Pearson's correlation test or Student's* t* test to determine the effects of clinical or echocardiographic parameters on exercise time. Multivariate linear regression analyses were conducted for factors that were significant in the univariate analysis to examine their independence. For further discussion about the distribution of these data and the result, we categorized the individuals in tertiles according to the GAS3d and applied one-way Analysis of variance (ANOVA) for a trend across ordered groups. Post-hoc analysis using the Scheffé test was done to evaluate the *P* values for each pair of comparison.

We defined METs < 8 as impaired exercise capacity. Multivariate logistic analysis was used to identify independent factors for impaired exercise capacity. Receiver operating characteristic (ROC) curve analysis was used to calculate the area under curve (AUC) and computed to determine optimal strain cutoff values, which derived from the Youden index, in individuals with preserved or impaired exercise capacity. The incremental value of GAS on E/e' for prediction of exercise capacity was assessed by Chi-Square statistics.

All data are presented as mean ± standard deviation or number (%). All statistical analyses were performed using SPSS 17.0 for Windows (SPSS Institute, Chicago, IL, USA), and a *P* value of <0.05 was regarded as significant.

## Results

### Clinical Characteristics

Our final study population comprised 94 participants (52.2 ± 11.7 years, 62.8% male) after the exclusion of 2 subjects because of insufficient image quality for analysis. In total, 19, 12, and 33 participants had hypertension, diabetes, and hyperlipidemia, respectively; thirteen were smokers. The detailed characteristics of the study population are listed in Table 1.

### Association between Exercise Time and Clinical Characteristics

The exercise time of the participants was 499.9 ± 145.6 seconds, and their MET was 10.0 ± 2.5 (Table 1). Exercise time was significantly associated with age (r = -0.469, *P* < 0.001) and systolic blood pressure (SBP; r = -0.258, *P* = 0.012), was higher in men (531.9 ± 142.8 vs. 446.0 ± 133.8 sec; *P* = 0.005), lower in those with hypertension (435.5 ± 158.3 vs. 516.2 ± 138.6 sec; *P* = 0.030), and higher in those who smoke (620.1 ± 52.7 vs. 480.6 ± 146.6 sec; *P* < 0.001; Table 2). The higher exercise times in smokers may be attributed to all 13 smokers being men and younger (52.6 ± 12.0 years vs. 49.6 ± 9.5 years, *P* =0.394).

### Association between Exercise Time and Echocardiographic Parameters

The echocardiographic parameters significantly associated with exercise time were early mitral filling velocity to average mitral annulus velocity ratio (E/e'; r = -0.403, *P* < 0.001), GRS3d (r = 0.208, *P* = 0.044), and GAS3d (r = -0.662, *P* < 0.001) but not GLS2d (r = -0.076, *P* = 0.468) and LVEF whenever M-mode (r = 0.158, *P* = 0.128), 2D (r = 0.104, *P* = 0.317), or 3D echocardiography (r = 0.047, *P* = 0.655) was employed (Table 2).

### Independent Factors for Exercise Time

**Figure 1:**
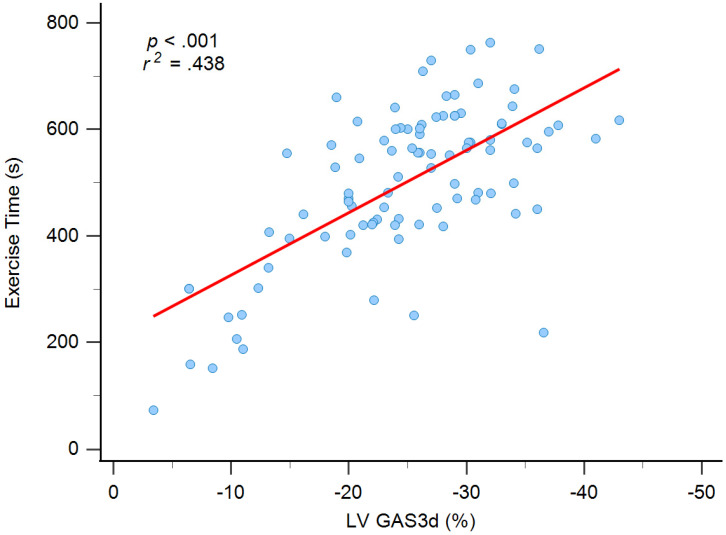
Graph showing moderate correlation between exercise time and left ventricular global area strain derived from 3D speckle-tracking echocardiography (LV GAS3d).

We included six statistically significant factors in the univariate analysis into a multivariate regression analysis to determine independent factors for exercise time. Two variables, hypertension and GRS3d, were excluded because high correlation with SBP and GAS3d, respectively. In the model of multivariate regression analysis, we observed that older age (ß = -0.194, *P* = 0.024), and women (ß = -0.172, *P* = 0.029) had lower exercise times, and GAS3d levels (ß = -0.486, *P* < 0.001) were significantly associated with exercise time (Table 3 and Figure 1). Significant association was still noted between smoking and exercise time (ß = 0.819, *P* = 0.047) but the *P* value was near 0.05.

The individuals were divided into three groups according to the GAS3d tertiles: first tertile (GAS3d > -22.31%; n = 31), second tertile (-22.31% ≥ GAS3d ≥ -29.0%; n = 31), and third tertile (GAS3d < -29.0%; n = 32). We applied one-way ANOVA and it showed this trend for exercise time among tertile groups reached statistical significance (first tertile to third tertile; 395.8 ± 146.8 seconds vs. 432.0 ± 158.8 seconds vs. 545.2 ± 119.3 seconds, *P* < 0.001; Supplementary Table 1 and Supplementary Figure 1). We could conclude that there were significant differences among groups and it meant the regression line of GAS3d versus exercise time was not polarized by the few points in the lower left portion of Figure 1. For each pair of comparison, we performed post hoc analysis and it revealed statistically significant difference between first tertile versus the other tertiles (both *P* value < 0.001). Besides exercise time, we found that age (first tertile to third tertile; 59.0 ± 10.4 years vs. 49.4 ± 10.6 years vs. 48.3 ± 11.3 years, *P* < 0.001), SBP (141.7 ± 21.0 mmHg vs. 133.4 ± 18.2 mmHg vs. 125.6 ± 17.7 mmHg, *P* = 0.005), diastolic blood pressure (DBP; 85.8 ± 11.4 mmHg vs. 85.0 ± 12.6 mmHg vs. 78.1 ± 12.0 mmHg, *P* =0.023), body mass index (BMI; 23.1 ± 3.2 kg/m^2^ vs. 24.6 ± 2.8 kg/m^2^ vs. 26.0 ± 3.3 kg/m^2^, *P* =0.002) and LV mass index (LVMI; 108.9 ± 44.1 g/m^2^ vs. 85.9 ± 23.5 g/m^2^ vs. 85.5 ± 27.1 g/m^2^, *P* = 0.006) were significantly associated between GAS3d tertile groups, but not E/e' (8.8 ± 3.3 vs. 7.86 ± 2.3 vs. 7.38 ± 2.9, *P* = 0.129) and LVEF whenever M-mode (69.6 ± 8.0 vs. 72.5 ± 7.5 vs. 70.5 ± 6.0, *P* = 0.276), 2D (63.1 ± 8.1 vs. 67.7 ± 9.5 vs. 67.6 ± 9.7, *P* = 0.317), or 3D echocardiography (60.3 ± 6.9 vs. 60.0 ± 5.8 vs. 62.2 ± 5.7, *P* = 0.311) was employed (Supplementary Table 1). GAS3d was an unique parameter in association with exercise time and our some clinical characteristics.

### Independent Factors for Exercise Capacity

Eighteen (19.1%) participants exhibited impaired functional capacity (MET < 8). Age (impaired vs. preserved exercise capacity; 62.7 ± 10.1 years vs. 49.7 ± 10.6 years, *P* < 0.001), SBP (143.9 ± 17.2 mmHg vs. 131.0 ± 19.8 mmHg, *P* = 0.013), BMI (26.0 ± 3.9 kg/m^2^ vs. 24.3 ± 3.1 kg/m^2^, *P* = 0.045), presence of hypertension (36.8% vs. 14.7%, *P* = 0.028), E/e' (10.3 ± 3.5 vs. 7.5 ± 2.4, *P* < 0.001), GLS3d (-14.6 ± 4.7% vs. -16.7 ± 3.7%, *P* = 0.038), and GAS3d (-14.4 ± 8.5% vs. -27.2 ± 6.0 %, *P* < 0.001) were significantly associated with impaired exercise capacity (Table 4). After multivariate logistic analysis with model 1 and model 2 to separately discuss GLS3d and GAS3d, it showed GAS3d (OR 1.24, 95% CI 1.10-1.39, *P* < 0.001) was an independent factor for impaired exercise capacity (Table 4).

**Figure 2:**
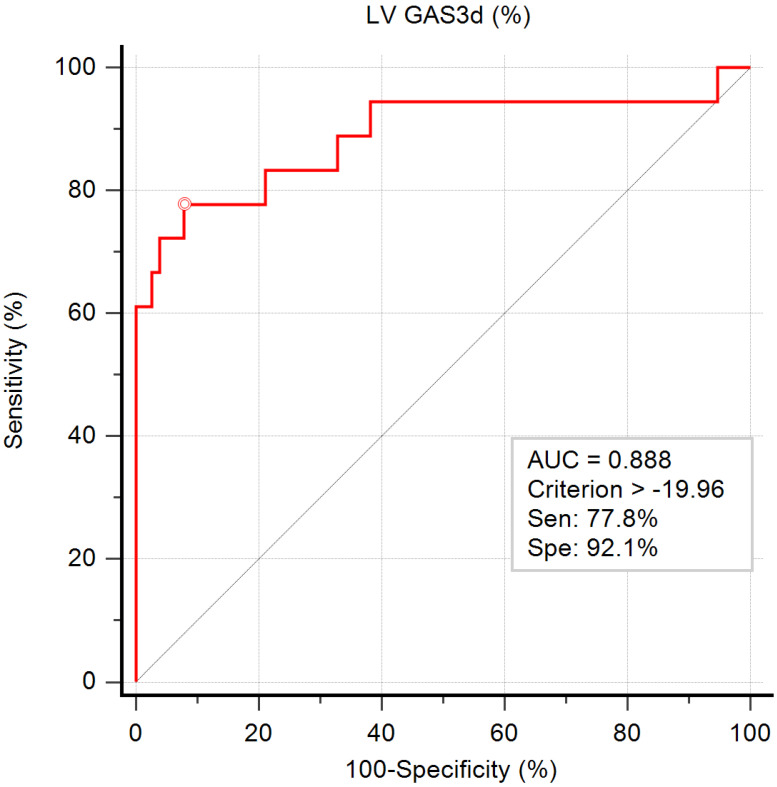
Graph showing the area under the curve (AUC) measured from receiver operating characteristic curve analysis for impaired exercise capacity by left ventricular global area strain derived from 3D speckle-tracking echocardiography (LV GAS3d) and the best cutoff point, derived from Youden index, with the highest sum of sensitivity and specificity.

The ROC curve for the prediction of impaired exercise capacity is illustrated in Figure 2. The AUC of GAS3d was 0.888, indicating that GAS3d is a suitable parameter for predicting impaired exercise capacity. According to the results of the ROC curve, we applied -19.96% of GAS3d as a cutoff point and the sensitivity and specificity were 77.8% and 92.1%, respectively.

### Incremental predictive value of GAS3d on E/e' for Exercise Capacity

**Figure 3:**
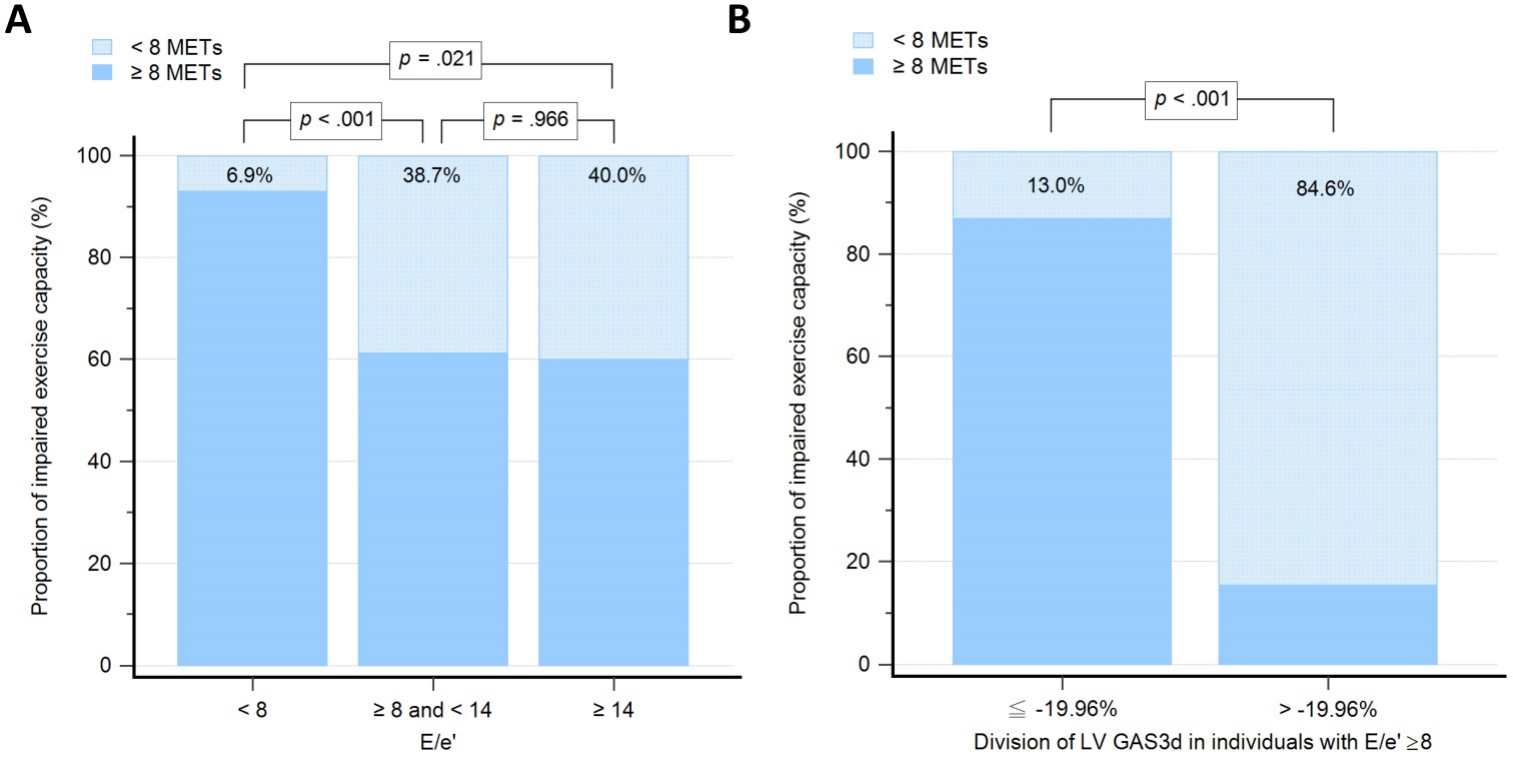
Graph showing the proportion of individuals with impaired exercise capacity among different groups. **3A:** Groups, as determined by 8 and 14 of early mitral filling velocity to average mitral annulus velocity ratio (E/e'). **3B:** Groups with early mitral filling velocity to average mitral annulus velocity ratio (E/e') more than and equal to 8, which further divided according to the -19.96% of left ventricular global area strain derived from 3D speckle-tracking echocardiography (LV GAS3d).

We grouped participants based on the values of E/e': low group (E/e' < 8; n = 58), medium group (8 ≤ E/e' < 14; n = 31), and high group (E/e' ≥14; n = 5). The proportion of individuals with impaired exercise capacity among E/e' groups were 6.9%, 38.7% and 40.0%, respectively (Figure 3A). The differences of proportion were statistically significant between low group versus medium group (*P* < 0.001) and low group versus high group (*P* = 0.021), but not medium group versus high group (*P* = 0.966). To examine whether the GAS3d could increase predictive ability on E/e' for impaired exercise capacity, we further divided participants whose E/e' ≥ 8 into two groups according to -19.96% of GAS3d, which derived from the Youden index (GAS ≤ -19.96% vs. GAS3d > -19.96; n = 23 vs. 13). The proportion of individuals with impaired exercise capacity was 13.0% and 84.6%, respectively (*P* < 0.001; Figure 3B).

## Discussion

The major finding of this study was that LV GAS3d was significantly associated with exercise time, as well as being an independent predictor of impaired exercise capacity. The cut-off value of -19.96% LV GAS3d represented the best discriminative value. LV GAS3d had an incremental value to E/e' in exercise capacity prediction.

Exercise capacity is a strong predictor of mortality not only in patients with cardiovascular disease but also among healthy individuals 29, 34. A prospective study 29 of 6213 consecutive men referred for TMT reported that every 1-MET increase in treadmill performance was associated with a 12% improvement in survival. The study also revealed that exercise capacity expressed as a percentage of the age-predicted value was not superior to the absolute peak exercise capacity in terms of predicting survival. A MET of 8 is a reliable cutoff value to identify individuals with preserved or impaired exercise capacity 25.

Because not all patients can undergo the treadmill test to evaluate exercise capacity, other determinants for evaluating exercise capacity are vital, especially for those who are too frail or are physically unfit to exercise long enough. An association between echocardiographic parameters and exercise capacity can be helpful in researching both physiological and potential clinical applications. LV systolic function is already an established prognostic marker. However, in a large cross-sectional study 35 of patients referred for exercise echocardiography and not limited by ischemia, LVEF variation within the broad range of normal values was not associated with exercise capacity. Similarly, our study indicated that LVEF, by whatever methods, failed to identify impaired exercise capacity, which indicates that LVEF is not a suitable echocardiographic parameter for evaluating exercise capacity.

Previous studies have reported STE as being superior to LVEF in detecting early reduction in myocardial systolic function 36 and as having prognostic value 37, 38. GLS2d has also been reported to be independently associated with exercise capacity in patients with heart failure with either reduced or preserved ejection fraction 39. It demonstrated a relationship between exercise capacity and resting myocardial function. It also confirmed EF's low sensitivity and limited quantitative value in individuals with heart failure with preserved EF. GLS2d and GLS3d were not independently associated with exercise time or capacity in our study. Only GAS3d was significantly associated with exercise time and was an independent predictor for impaired exercise capacity. Therefore, in individuals undergoing health check-up, GAS3d is superior to other myocardial function parameters in identifying patients with impaired exercise capacity.

Recent studies indicated that GAS3d with higher correlations with LV EF and cardiac output than the other strains 4, 40 and only GAS3d could differentiate patients with stage A heart failure from healthy adults 41. The novel 3D deformation parameter GAS3d is defined as the percentage change in the endocardial area at LV end-systole from its original area at end-diastole 3. Because area is the product of length and width, GAS3d can integrate the regional consequences of deformation in longitudinal and circumferential dimensions 42. By the law of volume conservation, radial strain also could be calculated from area strain. GAS3d was a comprehensive parameter of myocardial systolic deformation in all three dimensions and was obtained only from 3D STE. Therefore, these resulted in GAS3d could be the most sensitive parameter for detecting occult myocardial dysfunction in healthy population.

Although several studies have demonstrated the utility of 3D STE in various clinical scenarios, the clinical implications of GAS3d are currently being explored. The association between GAS3d and blood pressure has previously been reported in individuals with high-normal blood pressure 43 and native hypertensive patients 44. Recent studies have also revealed that GAS3d is a sensitive marker of subendocardial damage in adults after optimal repair of aortic coarctation 45 and tetralogy of Fallot 46. Furthermore, GAS3d was independently associated with increased risk of death or heart failure following acute myocardial infarction 47. Our study is the first study showing the significant association of LV GAS3d with exercise capacity in population who were free from symptoms and had no known structural heart disease, angiographically documented coronary artery disease, or atrial fibrillation before examinations. This result implicated that GAS3d might have potential role in identifying heart failure with preserved EF.

In our study, 22 people had positive ischemic result of TMT and it indicated an increased risk of coronary events and death for them. If we excluded those from our analysis, GAS3d was still significantly associated with exercise time, MET and exercise capacity (*P* < 0.001, *P* =0 .001, *P* = 0.018, respectively; Supplementary Table 2 and 3). GAS3d is a strong predictor of exercise capacity even in a low-risk population without positive TMT and it can be a useful parameter for health check-up individuals.

During exercise, the diastole is shortened, which causes a subsequent increase in filling pressures and decrease in the maximal capacity. Several studies have demonstrated a strong correlation between tissue Doppler of the mitral annulus and maximal exercise capacity 48, 49. Values for E/e' less than 8 usually indicated normal LV filling pressures and values more than 14 had high specificity for increased LV filling pressures 31. We used 8 and 14 of E/e' to divide participants into three groups. Notably, the proportion of individuals with impaired exercise capacity in low E/e' group was measured as 6.9% which was relatively lower compared with the other two groups. However, there was no statistically significant difference between medium and high E/e' groups (38.7% vs. 40.0%, respectively; *P* = 0.966). Thus, E/e' was not a useful indicator for identifying impaired exercise capacity in individuals with E/e' more than and equal to 8. Other studies 39, 50 have suggested that systolic and diastolic functions are closely linked and that both phases contribute to overall cardiac performance. By applying -19.96% of LV GAS3d on participants with E/e' more than or equal to 8, the proportion of individuals with impaired exercise capacity could be clearly distinguished between preserved and impaired GAS3d (13.0% vs. 84.6%, respectively; *P* < 0.001). Overall, LV GAS3d is a powerful parameter and exerts incremental value on E/e' to predict exercise capacity.

## Clinical Implications

In the real world, certain limitations exist for TMT and pharmacologic stress test, such as physical disabilities and drug-induced arrhythmia or hypotension. Thus, investigating a novel imaging tool for evaluating global cardiac performance as well as exercise capacity is helpful. Resting echocardiography is more readily available as a bedside clinical tool. Our study revealed that LV GAS3d derived from 3D STE is a comprehensive parameter of myocardial systolic deformation and is highly sensitive to early functional abnormalities in populations with impaired exercise capacity. Our study population included individuals with low risk and in stage A heart failure criteria according to ACC/AHA heart failure classification allows our results to be generalizable and applicable to a real-world clinical population in whom were mostly referred for evaluation. By using LV GAS3d, we can detect subtle myocardial dysfunctions and then assess any unexplored, and possibly substantial, risk of cardiac mortality and morbidity in the early phase. With potential widespread clinical use, LV GAS3d may have vital implications for the primary and secondary prevention of the leading causes of disability and death, especially heart-related disease.

## Limitations

We recognize several limitations in this study. First, STE is highly dependent on image quality. Several conditions, such as poor acoustic windows, arrhythmia, and severe LV chamber dilation, can contribute to suboptimal image quality. Poor acoustic windows after surgery or due to unusual heart positions are common in clinical scenarios. Arrhythmia may generate unreliable analysis because of substantial beat-to-beat variability. Although 3D STE with single-beat full-volume acquisition is valuable in such conditions, severe LV chamber dilation may still result in failure to include the entire LV myocardium for 3D analysis. Second, the study population was small and from a single medical center. Because smaller sample sizes become decreasingly representative of the entire population, future studies with larger sample sizes are required to verify our results. We included only participants without structural heart disease who were undergoing health check-up. Hence, the study results are likely not applicable to all cardiac disease or certain disorders precluding stress test, such as inability to exercise due to extreme obesity or other physical or mental impairments. Third, exercise capacity is the output of overall function of the cardiovascular, respiratory, and musculoskeletal systems. In our study, four individuals with impaired exercise capacity had GAS3d less than and equal to -19.96%. Other possible etiologies, contributed to impaired exercise capacity, could have played important roles in predicting mortality in these participants. Fourth, LV GAS3d was significantly associated with exercise capacity but not directly associated with clinical prognosis. Large prospective longitudinal studies are warranted to clarify the role of LV GAS3d in prognosis.

## Conclusions

LV GAS3d, uniquely derived by resting 3D STE, was a major predictor of exercise capacity in a health check-up population undergoing TMT. Deformation imaging from 3D STE has considerable potential to detect myocardial damage at a subclinical stage, beyond LVEF or 2D STE. The clinical application of this parameter merits further study.

## Highlights

- GAS is a novel 3D STE parameter to detect occult LV dysfunction.
- GAS is independently associated with exercise time.
- GAS adds incremental value to the E/e' in predicting exercise capacity.

## Supplementary Material

Supplementary figure and tables.

## Figures and Tables

**Table 1:** Baseline patient, echocardiographic, and treadmill characteristics

Variable	Values
Men	62.8% (59/94)
Age (y)	52.2 ± 11.7
SBP (mmHg)	133.5 ± 19.9
DBP (mmHg)	82.9 ± 12.4
Heart rate (beats/min)	80.7 ± 12.0
BMI (kg/m^2^)	24.3 ± 3.3
Hypertension	20.2% (19/94)
Diabetes mellitus	12.8% (12/94)
Hyperlipidemia	35.1% (33/94)
Smoking	13.8% (13/94)
E/e'	8.0 ± 2.9 (3.6 to 20.0)
LVEF (%), M-mode	70.8 ± 7.2 (53 to 90)
LVEF (%), 2D	66.1 ± 9.3 (49 to 86)
LVEF (%), 3D	60.8 ± 6.2 (42 to 75)
LV end-systolic volume (ml), 3D STE	30.2 ± 11.4 (15 to 68)
LV end-diastolic volume (ml), 3D STE	75.0 ± 23.1 (28 to 163)
LVMI (g/m^2^)	91.5 ± 33.7 (35 to 214)
GLS2d (%)	-19.2 ± 3.1
GLS3d (%)	-16.3 ± 4.0
GCS3d (%)	-18.0 ± 5.6
GAS3d (%)	-24.7 ± 8.2
GRS3d (%)	41.2 ± 16.4
Exercise time (second)	499.9 ± 145.6 (73 to 763)
METs	10.0 ± 2.5 (3.4 to 14.7)
Positive ischemic result, TMT	23.4% (22/94)
Negative ischemic result, TMT	69.1% (65/94)
Indeterminate ischemic result, TMT	7.4% (7/94)

Data are expressed as mean ± SD (range) or percentages. y = years old, s= seconds, SBP = systolic blood pressure, DBP = diastolic blood pressure, BMI = body mass index, LVMI = LV mass index.

**Table 2:** Association of clinical and echocardiographic parameters on exercise time

Variable	Exercise time(s)	*r*	*P*
Age		-.469	<.001*
SBP		-.258	.012*
DBP		-.114	.273
Heart rate		-.100	.337
BMI		-.133	.201
E/e'		-.403	<.001*
LVEF, M-mode		.158	.128
LVEF, 2D		.104	.317
LVEF, 3D		.047	.655
LVMI		-.203	.058
GLS2d		-.076	.468
GLS3d		-.095	.364
GCS3d		.061	.560
GAS3d		-.662	<.001*
GRS3d		.208	.044*
Gender			
Male (59)	531.9 ± 142.8		.005*
Female (35)	446.0 ± 133.8		
**Hypertension**			
Present (19)	435.5 ± 158.3		.030*
Absent (75)	516.2 ± 138.6		
**Diabetes mellitus**			
Present (12)	484.1 ± 110.8		.689
Absent (82)	502.2 ± 150.4		
**Hyperlipidemia**			
Present (33)	522.6 ± 122.4		.268
Absent (61)	487.6 ± 156.3		
**Smoking**			
Present (13)	620.1 ± 52.7		<.001*
Absent (81)	480.6 ± 146.6		

*P < .05, statistically significant;* S*, second.

**Table 3:** Multivariate linear regression analysis of significant clinical and echocardiographic parameters associated with exercise time

Variable	*ß* (95% CI)	*P*
Age	-.194 (-4.525, -.319)	.024*
Gender (female)	-.172 (-97.942, -5.351)	.029*
SBP	-.008 (-1.266, 1.153)	.926
Smoking	-.154 (.819, 128.050)	.047*
E/e'	-.102 (-14.524, 3.042)	.255
GAS3d	-.486 (-11.545, -5.675)	<.001*

*CI*, confidence interval; *P < .05, statistically significant.

**Table 4:** Comparison of clinical and echocardiographic characteristics between preserved (MET ≥ 8) and impaired exercise capacity (MET < 8)

Variable	Univariate analysis			Multivariate analysis			
				Model 1		Model 2	
	METs ≥ 8	METs < 8	*P*	OR (95% CI)	*P*	OR (95% CI)	*P*
Patient number (n)	76	18					
Age (y)	49.7 ± 10.6	62.7 ± 10.1	<.001*	1.12 (1.04-1.21)	.003*	1.09 (0.99-1.19)	.071
SBP (mmHg)	131.0 ± 19.8	143.9 ± 17.2	.013*	1.01 (0.97-1.04)	.772	1.00 (0.96-1.05)	.944
DBP (mmHg)	82.6 ± 12.9	84.3 ± 10.5	.595				
Heart rate (beats/min)	80.6 ± 11.7	80.9 ± 13.5	.921				
BMI (kg/m^2^)	24.3 ± 3.1	26.0 ± 3.9	.045*	1.18 (0.97-1.44)	.099	1.02 (0.80-1.31)	.861
E/e'	7.5 ± 2.4	10.3 ± 3.5	<.001*	1.18 (0.95-1.47)	.131	1.12 (0.83-1.51)	.452
LVMI (g/m^2^)	87.4 ± 28.7	108.9 ± 46.7	.085				
LVEF (%), M-mode	71.5 ± 6.8	68.1 ± 8.5	.069				
LVEF (%), 2D	66.3 ± 9.4	65.3 ± 9.0	.674				
LVEF (%), 3D	61.2 ± 6.3	59.0 ± 5.5	.168				
GLS2d (%)	-19.4 ± 2.9	-18.0 ± 3.6	.075				
GLS3d (%)	-16.7 ± 3.7	-14.6 ± 4.7	.038*	1.07 (0.91-1.26)	.406		
GCS3d (%)	-18.1 ± 5.4	-17.8 ± 6.3	.854				
GAS3d (%)	-27.2 ± 6.0	-14.4 ± 8.5	<.001*			1.24 (1.09-1.39)	<.001*
GRS3d (%)	42.4 ± 16.7	36.0 ± 14.2	.136				
Male (n, %)	50 (53.2%)	9 (9.6%)	.213				
Hypertension (n, %)	12 (12.7%)	7 (7.4%)	.028*				
Diabetes mellitus (n, %)	10 (10.6%)	2 (2.1%)	.815				
Hyperlipidemia (n, %)	30 (31.9%)	3 (3.2%)	.068				
Smoking (n, %)	13 (13.8%)	0 (0%)	.059				

*OR*, odds ratio; *CI*, confidence interval; *P < .05, statistically significant.

## Abbreviations

2D: Two-dimensional
3D: Three-dimensional
E/e': Early mitral filling velocity to average mitral annulus velocity ratio
GAS: Global area strain
GCS: Global circumferential strain
GLS: Global longitudinal strain
GRS: Global radial strain
LV: Left ventricular
LVEF: Left ventricular ejection fraction
LVQ: Left Ventricular Quantification
MET: Metabolic equivalent of task
NYHA: New York Heart Association
STE: Speckle-tracking echocardiography
TMT: Treadmill exercise test
