# Supplementary material for: Association of left ventricular global area strain derived from resting 3D speckle-tracking echocardiography and exercise capacity in individuals undergoing treadmill exercise test
Source: Int J Med Sci. 2022 Sep 11;19(10):1576–85. doi: 10.7150/ijms.75781 (PMC9515695; doi:10.7150/ijms.75781)
Supplement: Supplementary file 1 — Supplementary figure and tables. [file ijmsv19p1576s1.pdf]

Supplementary Figure 1 Graph showing there is a significant linear trend for exercise time among tertiles of left ventricular global area strain derived from 3D speckle-tracking echocardiography (LV GAS3d)

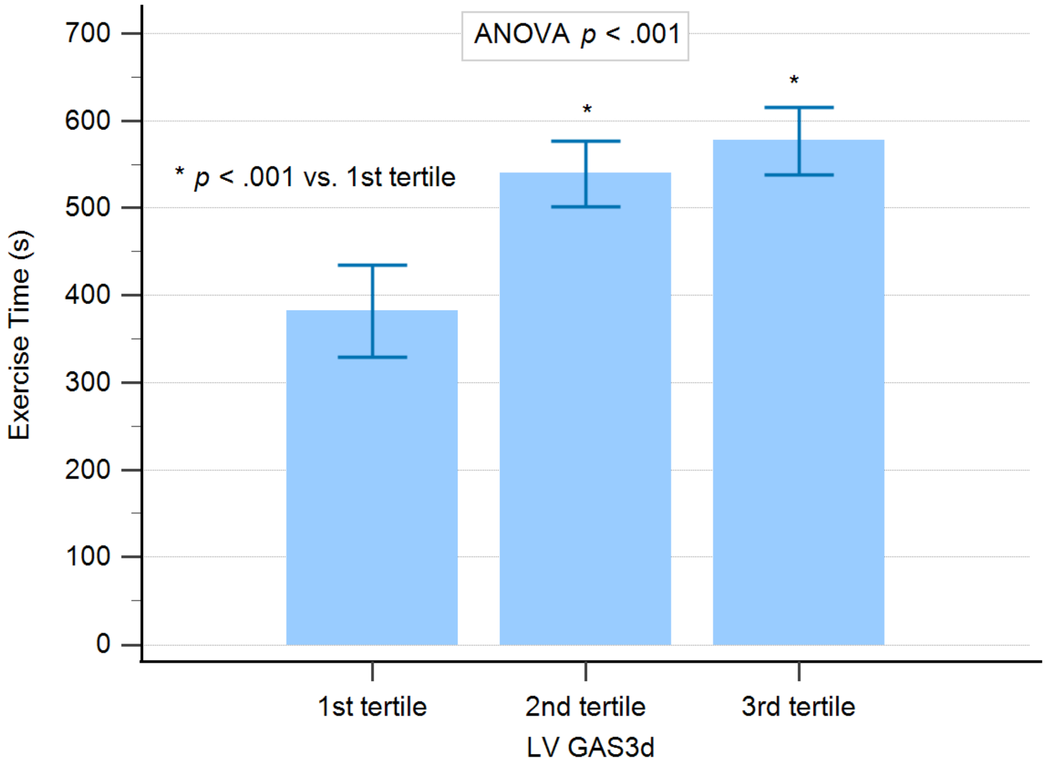

Supplementary Figure 1

Supplementary Table 1 Baseline characteristics by tertile of LV GAS3d

Variable	1st tertile	2nd tertile	3rd tertile	*P*
	> -22.31%	-29.0%~-22.31%	< -29.0%	
Exercise time (s)	395.8 ± 146.8	432.0 ± 158.8	545.2 ± 119.3	<.001*
Age (y)	59.0 ± 10.4	49.4 ± 10.6	48.3 ± 11.3	<.001*
SBP (mmHg)	141.7 ± 21.0	133.4 ± 18.2	125.6 ± 17.7	.005*
DBP (mmHg)	85.8 ± 11.4	85.0 ± 12.6	78.1 ± 12.0	.023*
BMI (kg/m^2)	23.1 ± 3.2	24.6 ± 2.8	26.0 ± 3.3	.002*
LVMI (g/m^2)	108.9 ± 44.1	85.9 ± 23.5	85.5 ± 27.1	.006*
E/e’	8.8 ± 3.3	7.86 ± 2.3	7.38 ± 2.9	.129
LVEF, M-mode (%)	69.6 ± 8.0	72.5 ± 7.5	70.5 ± 6.0	.276
LVEF, 2D (%)	63.1 ± 8.1	67.7 ± 9.5	67.6 ± 9.7	.317
LVEF, 3D (%)	60.3 ± 6.9	60.0 ± 5.8	62.2 ± 5.7	.311

**P* < .05, statistically significant; *S*, second

Supplementary Table 2 Association of clinical and echocardiographic parameters on exercise time and METs in individuals without positive treadmill exercise test

Variable	Exercise time		MET	
	Univariate analysis *P*	Multivariate analysis *P*	Univariate analysis *P*	Multivariate analysis *P*
Age	<.001*	.224	<.001*	.147
SBP	.055		.066	
DBP	.075		.083	
Heart rate	.969		.352	
BMI	.016*	.903	.017*	.887
E/e’	<.001*	.342	.002*	.502
LVEF, M-mode	.322		.309	
LVEF, 2D	.504		.623	
LVEF, 3D	.774		.786	
LVMI	.124		.158	
GLS2d	.775		.823	
GLS3d	.312		.468	
GCS3d	.819		.886	
GAS3d	<.001*	<.001*	<.001*	.001*
GRS3d	.173		.177	
Gender	.012*	.078	.011*	.081
Hypertension	.002*	.423	.004*	.446
Diabetes mellitus	.456		.541	
Hyperlipidemia	.906		.824	
Smoking	.005*	.112	.004*	.080

*$P < .05$, statistically significant; MET = Metabolic equivalent of task

Supplementary Table 3 Comparison of clinical and echocardiographic characteristics between preserved (MET ≥ 8) and impaired exercise capacity (MET < 8) in individuals without positive treadmill exercise test

Variable	Univariate analysis			Multivariate analysis			
				Model 1		Model 2	
	METs ≥ 8	METs < 8	*P*	OR (95% CI)	*P*	OR (95% CI)	*P*
Patient number (n)	60	12					
Age (y)	49.8 ± 11.3	61.3 ± 11.0	.002*	1.08 (0.99-1.18)	.070	1.06 (0.96-1.17)	.281
SBP (mmHg)	129.0 ± 20.3	139.9 ± 12.3	.077				
DBP (mmHg)	82.0 ± 13.2	87.3 ± 9.82	.195				
Heart rate (beats/min)	81.1 ± 12.3	77.5 ± 10.1	.352				
BMI (kg/m^2)	24.0 ± 2.6	26.4 ± 4.2	.012*	1.24 (0.94-1.63)	.130	1.03 (0.71-1.50)	.868
E/e’	7.4 ± 2.4	10.2 ± 2.9	.001*	1.10 (0.82-1.49)	.529	1.05 (0.70-1.59)	.806
LVMI (g/m^2)	87.6 ± 30.4	101.7 ± 44.9	.201				
LVEF (%), M-mode	71.4 ± 7.1	68.3 ± 7.9	.177				
LVEF (%), 2D	66.3 ± 9.7	67.0 ± 9.0	.797				
LVEF (%), 3D	61.1 ± 5.9	59.5 ± 5.1	.380				
GLS2d (%)	-19.4 ± 2.6	-18.4 ± 3.5	.251				
GLS3d (%)	-16.7 ± 3.6	-13.8 ± 5.0	.019*	1.09 (0.89-1.32)	.407		
GCS3d (%)	-18.3 ± 4.5	-16.5 ± 6.2	.250				
GAS3d (%)	-27.7 ± 6.0	-14.8 ± 9.9	<.001*			1.20 (1.03-1.39)	.018*
GRS3d (%)	43.2 ± 17.0	40.1 ± 14.8	.561				
Male (n, %)	39 (65.0%)	5 (41.7%)	.133				

Hypertension (n, %)	7 (11.7%)	7 (58.3%)	<.001*	4.14 (0.81-21.2)	.087	2.67 (0.37-19.5)	.333
Diabetes mellitus (n, %)	7 (11.7%)	2 (16.7%)	.635				
Hyperlipidemia (n, %)	23 (38.3%)	3 (25.0%)	.383				
Smoking (n, %)	10 (16.7%)	0 (0%)	.130				

OR, odds ratio; *CI*, confidence interval; *P < .05, statistically significant
